# Immune Thrombocytopenia Relapse in Patients Who Received mRNA COVID-19 Vaccines

**DOI:** 10.2147/JBM.S396026

**Published:** 2023-04-14

**Authors:** Hana Qasim, Alaa Rahhal, Ahmed Husain, Abdelkarim Alammora, Khaled Alsa’ed, Ahmed Abdelghafar Masaad Alsayed, Baha Faiyoumi, Leen Maen AbuAfifeh, Mohammad Abu-Tineh, Awni Alshurafa, Mohamed A Yassin

**Affiliations:** 1 Hematology-Oncology Department, National Centre for Cancer Care & Research, Doha, Qatar; 2 Department of Internal Medicine, University of Missouri-Kansas City, Kansas City, MO, USA; 3 MSc Pharmacy Department, Hamad Medical Corporation, Doha, Qatar; 4 Infectious Disease Department, Communicable Disease Center, Hamad Medical Corporation, Doha, Qatar; 5 Internal Medicine Department, Hamad Medical Corporation, Doha, Qatar

**Keywords:** ITP, vaccine, relapse, COVID-19

## Abstract

**Background:**

Immune thrombocytopenia (ITP) is a blood disorder in which antibodies coating platelets cause platelet destruction in the spleen with a resultant low platelet count and an increased tendency for bleeding. Coronavirus disease 2019 (COVID-19) is an illness caused by SARS-CoV-2. Though pneumonia and respiratory failure are major causes of morbidity and mortality, multisystemic complications were identified, including hematological ones. Several ITP relapse cases post-mRNA SARS-CoV-2 vaccines have been reported, and different pathophysiological theories have been proposed.

**Purpose:**

The objective of this study is to identify the causal relationship between mRNA COVID-19 vaccines and ITP relapse, to highlight the longer-term effect of these vaccines on the platelet count more than 6 months after receiving the vaccine, and to identify if there is a statistical difference between Comirnaty and Spikevax vaccines on ITP relapse rate.

**Patients and Methods:**

In this retrospective study, 67 patients with known ITP were followed before and after receiving the mRNA COVID-19 vaccine. The follow-up parameters included platelet counts when available and bleeding symptoms. All patients were adults over 18 years old, with no other identified causes of thrombocytopenia. Forty-seven patients received the Comirnaty vaccine, and 20 patients received the Spikevax vaccine.

**Results:**

Data analysis showed 6% ITP relapse in the first 3 months, and a 10% relapse rate 3–6 months after receiving one of the mRNA COVID-19 vaccines, with no statically significant difference between the two vaccines.

**Conclusion:**

mRNA COVID-19 vaccines increase the risk of ITP relapse and can lead to a prolonged reduction in platelet count in a proportion of ITP patients, with no statistically significant difference between Comirnaty and Spikevax vaccines.

## Introduction

Immune thrombocytopenic (ITP) is a syndrome in which platelets become coated with autoantibodies to platelet membrane antigens. In patients with ITP, the mononuclear macrophage system of the spleen is responsible for removing platelets, and with incomplete compensation from the bone marrow, this leads to a decrease in circulating platelets count. This proposition is supported by the prompt improvement of a significant proportion of patients after IVIG therapy and splenectomy.^1^,^2^ Patients can present with bruising, epistaxis, and mucosal bleeding, with a minority of cases developing major bleeding complications such as gastrointestinal tract bleeding and intracranial hemorrhage. The mRNA COVID vaccine is considered generally safe, with millions of people receiving the vaccine with no serious adverse effects, nevertheless, multiple complications, especially autoimmune phenomena have been reported so far,^3^ including but not limited to myocarditis, Guillain-Barre syndrome, autoimmune hepatitis, arthritis, and hematological complications like thrombotic thrombocytopenic purpura and immune thrombocytopenia purpura. Most reported ITP cases presented with petechia, bruising, and mucosal bleeding in the first few weeks after receiving the vaccine;^4^,^5^ and showed improvement with conventional steroid treatment. Nevertheless, recommendations for ITP patients to receive mRNA COVID vaccine are still under discussion. Few retrospective and prospective studies on ITP patients have shown an increased relapse rate after the COVID-19 vaccine.^6^,^7^

## Methods

This retrospective study was conducted to check platelet counts in 67 patients known to have immune thrombocytopenia purpura, who are >18-years-old, and not known for any other causes of thrombocytopenia (including congenital thrombocytopenia, malignancy, systemic lupus erythematosus, antiphospholipid syndrome). The platelet count was identified at baseline before receiving the vaccine and within 3 months, 6 months, and after 6 months after receiving the vaccine.

For Comirnaty (Pfizer) vaccine, the second dose was given 21 days after the first dose. For Spikevax (Moderna), the second dose was given 28 days after the first dose.

ITP relapse was defined as a drop in platelet counts to less than 30×10^9^/L with bleeding symptoms or a need to use rescue therapy.

This study aims to demonstrate the causal relationship between ITP relapse in known ITP patients and the mRNA COVID vaccine and to evaluate the long-term effect of the vaccine on ITP patients >6 months after receiving the second dose of the vaccine.

### Data Collection

Data were accessed from the existing medical records and were extracted and analyzed.

The selected clinical and pathological data were obtained from the patient charts and/or database. This data includes demographic data diagnosis, lab results and received treatment.

Data was only accessed by a specific username and password and was stored in the hospital computer.

### Statistical Analysis

**Figure 1:**
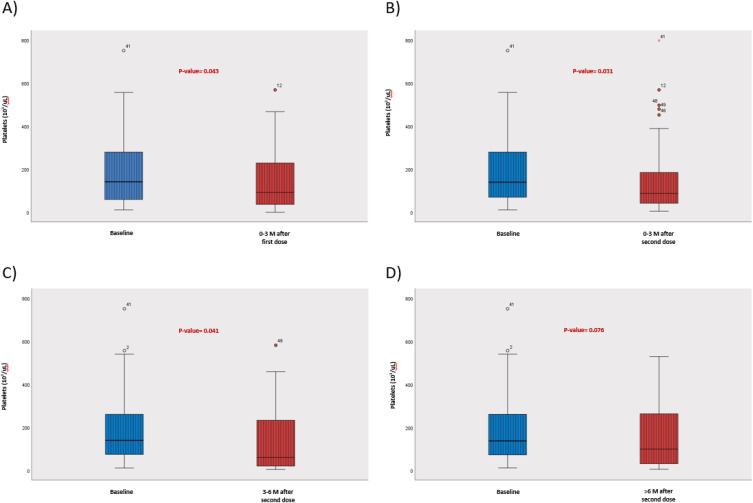
(**A-D**) The change in platelets after receiving COVID-19 vaccines among patients with ITP.

**Figure 2:**
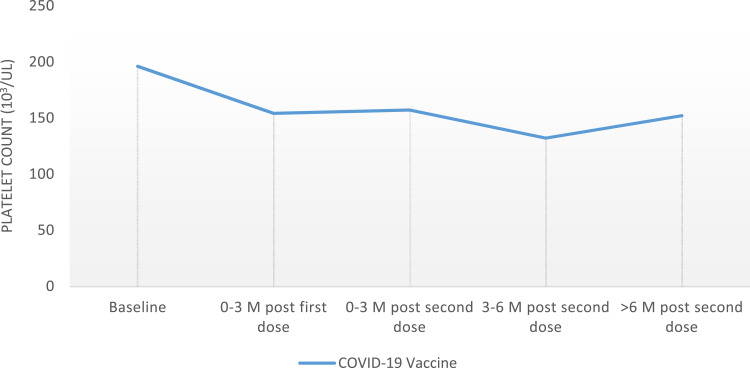
The trend of platelets after receiving COVID-19 vaccines among patients with ITP.

Data analyses (Figures 1 and 2) were performed using the Statistical Package for Social Sciences program version 28.0 (IBM SPSS Statistics for Windows; IBM Corp, Armonk, NY). Descriptive statistics were reported in the form of frequencies and percentages for categorical variables and mean ± standard deviation (SD) for continuous variables. The change in platelets after receiving COVID-19 vaccines was analyzed using the paired-sample *t*-test. Student’s *t*-test was used to compare the platelet counts between different vaccines. A *p*-value of <0.05 was used to indicate statistical significance.

## Results

### Patient Characteristics (Table 1)

We included 67 patients (Table 1), 32 (47.8%) males and 35 (52.2%) females, aged 40±13 years. Forty-sevenj (70.1%) patients received Comirnaty (Pfizer) COVID vaccine and 20 (29.9%) patients received Spikevax (Moderna) COVID vaccine. All patients received two doses of the vaccine as scheduled with no delay. Twenty-eight (41.8%) patients received prior treatment of Eltrombopag, two (3%) received prior Romiplostim, and 19 (28.4%) patients received prior treatment of Rituximab.

**Table 1:** Baseline Characteristics of Patients with ITP Who Received COVID-19 Vaccines (N=67)

Characteristic	n (%)
Age (years)	40±13
Gender	
Male	32 (47.8)
Female	35 (52.2)
Platelets (10^3^/µL)	196±154
COVID-19 infection	21 (31.3)
Platelets after COVID-19 infection	213±187
Vaccine	
Comirnaty (Pfizer)	47 (70.1)
Spikevax (Moderna)	20 (29.9)
ITP medications	
Steroids	3 (4.5)
Rituximab	19 (28.4)
Romiplostim	2 (3)
Eltrombopag	28 (41.8)

### COVID-19 Vaccination Effect on Platelet Count (Figures 1 and 2)

Data analysis showed a relapse rate of 9% (6 patients) in the first 3 months after the mRNA COVID-19 vaccine, 15% (10 patients) in 3–6 months, and a 12% (8 patients) relapse rate more than 6 months after receiving the vaccine (Table 2). The trend in absolute platelets count is illustrated in Table 3. There were no relapse cases discovered between the first and second doses of both vaccines. Data analysis also showed a significant platelets drop of >50% in eight (12%) patients, who did not meet the criteria for ITP relapse within the first 6 months after the vaccine, and four (6%) patients had a long-term effect with a >50% reduction in platelets count more than 6 months after receiving the vaccine (Table 2).

**Table 2:** Outcomes of Patients with ITP Who Received COVID-19 Vaccines (N=67)

Outcome	N (%)
ITP relapse within 3 months of vaccination	6 (9)
ITP relapse within 6 months of vaccination	10 (15)
ITP relapse within >6 months of vaccination	8 (12)
25–50% reduction in platelets within 6 months of vaccination	6 (9)
25–50% reduction in platelets >6 months of vaccination	3 (4.5)
>50% reduction in platelets within 6 months of vaccination	8 (12)
>50% reduction in platelets >6 months of vaccination	4 (6)

**Table 3:** Trend of Platelets After Receiving COVID-19 Vaccines Among Patients with ITP (N=67)

Interval	Platelets Before Vaccine	Platelets After Vaccine	*P*-value
0–3 months post first dose	196±154	154±152	0.043
0–3 months post second dose	196±154	157±183	0.031
3–6 months post second dose	196±154	132±152	0.041
>6 months post second dose	196±154	152±155	0.076

**Figure 3:**
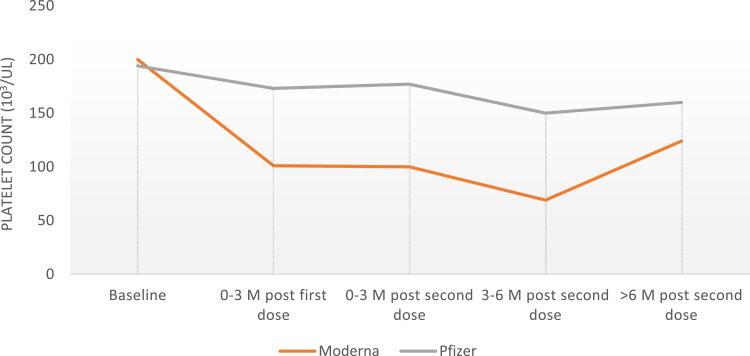
The trend of platelets after receiving Comirnaty (Pfizer) and Spikevax (Moderna) COVID-19 vaccines among patients with ITP.

A less dramatic effect on platelet count with a 25–50% drop was observed in 9% of patients within 6 months, and 4.5% more than 6 months after the vaccine. Data analysis did not show a significant difference in ITP relapse cases or platelet number trend between Comirnaty and Spikevax vaccines (Table 4; Figure 3). Of note, two patients had a persistently low platelet count after receiving the vaccine, despite standard ITP treatment. Of the patients who had ITP relapse after the vaccine, five had a recorded COVID-19 infection, and none of them had evidence of relapse within 1 month of the infection.

**Table 4:** Platelets After Receiving COVID-19 Vaccines Among Patients with ITP (N=67)

Interval	Platelets After Comirnaty Vaccine	Platelets After Spikevax Vaccine	*P*-value
Before vaccination	194±159	200±145	0.886
0–3 months post first dose	173±155	101±137	0.189
0–3 months post second dose	177±200	100±112	0.133
3–6 months post second dose	150±158	69±112	0.120
>6 months post second dose	160±163	124±131	0.474

We studied the possible predictors of relapse, including gender, age, previous COVID-19 infection, and ITP treatment, using a logistic regression model, which showed no significant effect of any of these factors, as all patients are at the same risk of relapse regardless of these factors (Table 5).

**Table 5:** Predictors of Relapse

Factor	Odds Ratio	95% Confidence Interval	*P*-value
Male gender	1.3	0.4–4.3	0.651
Age (older than 40 years)	1.6	0.5–5.0	0.435
Comirnaty vaccine	0.6	0.2–2.1	0.442
COVID infection	1.5	0.5–5.1	0.502
Concurrent steroids	1.9	0.1–31.9	0.664
Concurrent TPO-RA	1.0	0.3–3.2	0.955
Rituximab treatment	0.6	0.1–2.7	0.525

## Discussion

In response to the urgent need for effective vaccines to stop the COVID pandemic, the World Health Organization listed both the Comirnaty (Pfizer/BioNTech) vaccine and Spikevax (Moderna) COVID-19 vaccine (mRNA 1273) for emergency use listing on December 31, 2020 and the April 30, 2021, respectively. Both vaccines are mRNA-based vaccines that encode SARS-CoV-2 spike protein and enhance immunogenicity against the virus.^8^,^9^ There were no major safety concerns related to the vaccines initially,^10^,^11^ but, over time, there has been increased recognition of post-mRNA SARS-CoV-2 vaccine immune phenomena,^12^,^13^ including immune thrombocytopenia.^3^,^14^ With the need to have clear recommendations for ITP patients who are planning to receive the COVID-19 vaccine, we conducted this retrospective study, which has shown an increased relapse rate and a drop in platelets count, which is not limited to the first few weeks after the vaccine, as was thought before. As ITP relapse cases were reported before with older vaccines,^15–17^ there are some proposed potential mechanisms of immune activation triggered by vaccines administration, which can apply to the COVID-19 vaccine, including molecular mimicry,^3–5^ where the immune cross-reactivity triggered by the similarity between certain vaccine components and specific human proteins causes the immune system to attack normal tissue proteins in a susceptible population. Another is by creating a hyper-inflammatory environment, with increased production of the pro-inflammatory cytokine, resultant oxidative stress, DNA damage in the mononuclear cells, and T-cell dysregulation. The hyperinflammatory environment in bone marrow might lead to megakaryocyte apoptosis and a decrease in platelet count.^18^,^19^ Though most patients with COVID-19 vaccine-induced relapses respond to first-line treatment with steroids and IVIG, refractory cases were reported.^20^ The new persistent and likely different autoimmune process, where newly developed antibodies targeting new platelet antigens induced by molecular mimicry, associated with megakaryocyte apoptosis, might explain the treatment resistance that some ITP patients showed after receiving the vaccine.^20^

A recent multicenter study in the Netherlands showed exacerbation of ITP occurred in 30 of the 218 ITP patients (14%) after COVID-19 vaccination, risk factors for ITP relapse in subgroup analysis included patients with baseline platelet count < 50×10^9^/L, patients on ITP treatment at the start of COVID-19 vaccination, and patients with younger age. It also demonstrated a 6.3% decrease in platelet count compared with baseline, 4 weeks after the second vaccination.^21^

We found a more significant drop in platelets count from the baseline in patients who did not meet the criteria for ITP relapse (Figure 2), with platelets drop of >50% in 12% of patients within the first 6 months after the vaccine, and 6% of patients had a long-term effect with >50% reduction in platelets count more than 6 months after receiving the vaccine. Additionally, our data analysis did not show increased risk related to age, gender, type of treatment received, or previous COVID-19 infection. This does not exclude the possibility of the effect of these factors but might be related to the smaller sample size in our study.

On further follow-up, two patients with baseline platelets of >100×10^9^/L had persistently low platelet counts after receiving the vaccine with platelets count <30×10^9^/L despite standard ITP treatment, and one of them had splenectomy eventually. We propose that the long-term effect on some patients exceeding the first few weeks after the vaccine and the newly-developed refractory cases might be related to new and persistent autoimmune processes to new antigens in the susceptible patient.

Though up to 85% of ITP patients respond to initial standard treatment with steroids,^22^ only 15% maintain this response over the next year. In one study where 281 ITP patients were followed for 60 months, 51.6% developed a relapse event, of whom the median time between response to relapse was 1.16 (0.03–96) months.

Second-line therapy such as thrombopoietin receptors agonists, Rituximab, or splenectomy is required in a good proportion of patients to maintain safe platelet levels. In one Canadian study of 789 patients with thrombocytopenia, 204 received second-line therapy for ITP.^23^ The relapse rate after the second-line therapy depends on the used modality of treatment. For example, in one study of 137 patients with chronic ITP, 32% of patients who were given rituximab achieved a complete remission that was sustained for 1 year, and 63% were still in remission after 5 years. Two-thirds to 80% of ITP patients remain in remission after splenectomy.^22^

There is no one clear criteria to predict if the patient with ITP is going to have a relapse, or how many relapses he is expected to have in a lifetime, but the temporal relationship between the COVId-19 vaccine and the ITP relapses in our study, and the observation that a significant number of patient developed a relapse within a year following the vaccine regardless of their treatment modality, or time of ITP diagnosis, suggest a causal relationship between mRNA COVID-19 vaccine and ITP relapse.

Based on the reported cases and the conducted observational studies, the current recommendations encourage ITP patients to receive the mRNA COVID-19 vaccine, given the low risk for relapse, and the complete response to the standard ITP treatment.^2^,^14^ For patients who are having ITP relapse, consideration of delaying the vaccine for patients in active ITP flare till further stabilizations is recommended.^24^

We believe in the importance of the COVID-19 vaccine in decreasing the number and severity of COVID-19 infection cases, which by far outweigh the risks for the general population, nevertheless, ITP patients might be the group of patients that deserve more discussion about the possibility of relapse after receiving the vaccine, with recommendations for more frequent follow-ups in an extended period for about a year following the COVID-19 vaccine.

## Conclusion

MRNA COVID-19 vaccines increase the risk of ITP relapse and can lead to a prolonged reduction in platelet count in a proportion of ITP patients, with no statically significant difference between Comirnaty and Spikevax vaccines. We recommend more frequent follow-up for patients with ITP for 1 year following the mRNA COVID-19 vaccines for early detection and treatment of any significant platelet drop.

## Study Limitations

This is a relatively small sample size, and identifying risk factors of ITP relapse in ITP patients and detecting the difference between Spikevax and Comirnaty vaccines needs a larger sample size. There is no control group, and the correlation depends on the temporal relationship between the vaccine and the ITP relapse. A longer follow-up duration to determine the vaccine’s longer-term effect on platelet count is needed. Measuring anti-platelet antibodies before and after receiving the vaccine and bone marrow biopsy in resistant cases might help in determining the pathophysiology of patients who become refractory to treatment after receiving the vaccine.
